# Corrosion and transformation of solution combustion synthesized Co, Ni and CoNi nanoparticles in synthetic freshwater with and without natural organic matter

**DOI:** 10.1038/s41598-021-87250-7

**Published:** 2021-04-12

**Authors:** Alexander Khort, Jonas Hedberg, Nanxuan Mei, Valentin Romanovski, Eva Blomberg, Inger Odnevall

**Affiliations:** 1 Division of Surface and Corrosion Science, Department of Chemistry, KTH Royal Institute of Technology, Stockholm, Sweden; 2 Center of Functional Nano-Ceramics, National University of Science and Technology “MISIS”, Moscow, Russia; 3 Surface Science Western, Western University, London, Canada; 4 Institute of General and Inorganic Chemistry, National Academy of Sciences of Belarus, Minsk, Belarus; 5 Division Bioscience and Materials, RISE Research Institutes of Sweden, Stockholm, Sweden; 6 AIMES—Center for the Advancement of Integrated Medical and Engineering Sciences at Karolinska Insitutet and KTH Royal Institute of Technology, Stockholm, Sweden; 7 Department of Neuroscience, Karolinska Institutet, 171 77 Stockholm, Sweden

**Keywords:** Wetlands ecology, Nanoparticles, Synthesis and processing

## Abstract

Pure metallic Co, Ni, and their bimetallic compositions of Co_3_Ni, CoNi, and CoNi_3_ nanomaterials were prepared by solution combustion synthesis. Microstructure, phase composition, and crystalline structure of these nanoparticles (NPs) were characterized along with studies of their corrosion and dissolution properties in synthetic freshwater with and without natural organic matter (NOM). The nanomaterials consisted of aggregates of fine NPs (3–30 nm) of almost pure metallic and bimetallic crystal phases with a thin surface oxide covered by a thin carbon shell. The nanomaterials were characterized by BET surface areas ranging from ~ 1 to 8 m^2^/g for the Ni and Co NPs, to 22.93 m^2^/g, 14.86 m^2^/g, and 10.53 m^2^/g for the Co_3_Ni, CoNi, CoNi_3_ NPs, respectively. More Co and Ni were released from the bimetallic NPs compared with the pure metals although their corrosion current densities were lower. In contrast to findings for the pure metal NPs, the presence of NOM increased the release of Co and Ni from the bimetallic NPs in freshwater compared to freshwater only even though its presence reduced the corrosion rate (current density). It was shown that the properties of the bimetallic nanomaterials were influenced by multiple factors such as their composition, including carbon shell, type of surface oxides, and the entropy of mixing.

## Introduction

Nanotechnology is a high-tech multi-billion industry that during the latest decades has penetrated numerous aspects of modern society. A significant portion of the nano-market (~ 85%) is made up of nanomaterials (NMs)^1^. One of the main factors for such great interest is their sometimes superior and unique functional properties in comparison with their bulk analogs^2^. This has stimulated the development of new types of NMs of improved characteristics. For instance, metal nanopowders are among the most popular materials e.g. gas sensors^3–5^, catalysts for various technological processes^6,7^, electronics^8^, energy generator and storage devices^9–11^. Metallic NMs are further increasingly used in environmental protection-related applications^12,13^, and are of great interest for biomedical applications^14–18^. Nanoparticles (NPs) of single-phase multicomponent metal alloys with a high configurational entropy of mixing (i.e. low- middle- and high-entropy alloys) have for instance shown to be excellent alternatives to Pt and Pd catalysts^19–21^ and graphene-polymetallic nanocomposites have found applications as supercapasitors^22^ and highly-effective sensors^23^. The improvement in functional properties, along with their thermal and corrosion resistance, is usually associated with the effect of entropy stabilization. However, from such a rapid and drastic growth in the production of NMs follows an evident risk of unforeseen hazards related to their environmental and human exposures. Several studies have been carried out to assess biological and environmental effects of NMs, including features such as dissolution and corrosion of different types of NPs that may be environmentally dispersed^24–29^. It has been shown that multiple factors influence the dissolution and corrosion properties of NPs^30^. Physico-chemical characteristics of NMs of different classes cannot be automatically used for read-across, not even for NMs within the same class. This is for example the case for the relatively high toxic potency of CoO NPs compared with Co_3_O_4_ showing non-genotoxic properties^31^. Potential adverse effects induced by an increasing variety of new types of NMs and morphologies are most possibly unknown as traditional risk assessment tools cannot keep up with their speed of market entry. A systematic approach and development of fundamental criteria for the assessment and prediction of major factors that influence the stability and safety of NMs are hence urgently requested^30^.

In this study, the corrosion and dissolution properties of cobalt (Co), nickel (Ni), and bimetallic Co_x_Ni_y_ NPs, obtained by solution combustion synthesis (SCS) have been investigated in synthetic freshwater (FW) with and without natural organic matter (NOM). The SCS technique, which is based on self-propagating high-exothermic redox reactions, is an advanced, popular, and relatively easy, method for NM production^32^. In short, solid phase products form during exothermic redox reactions between components of a precursor, metal nitrates (oxidizer), and an organic fuel (reductant) which are mixed at a molecular level in a solution. Due to this feature, combustion of SCS precursors occurs even in inert atmospheres and in vacuum and result in high-quality NMs with homogeneous crystal phases. The method has previously been used to produce NMs of different functional classes, including pure and multicomponent metal NMs^33–36^, as well as simple and complex oxides^37–39^. Since SCS is one of few techniques that could easily be scaled-up for industrial production of a broad class of NMs, it is important to study particle properties as well as corrosion and dissolution characteristics of SCS NPs in e.g. environmental media, the focus of this study.

## Experimental

### Synthesis

All chemicals, exploited for the synthesis were of analytical grade and used without further purification. Cobalt nitrate hexahydrate (Co(NO_3_)_2_·6H_2_O) and nickel nitrate hexahydrate (Ni(NO_3_)_2_·6H_2_O) were used as metal atom sources and oxidizers. Hexamethylenetetramine (C_6_H_12_N_4_, HMT) was used as organic fuel/reducer. Synthetic FW (NaHCO_3_—6.5 mg/L, KCl—0.58 mg/L, CaCl_2_·2H_2_O—29.4 mg/L, MgSO_4_·7H_2_O—12.3 mg/L)^40^ with and without the addition of Suwannee river NOM (FWN) (10 mg/L) were adjusted to ~ pH 6.2, for the electrochemical, corrosion and dissolution investigations.

Co, Ni and binary Co–Ni NPs were obtained via the SCS redox reaction of metal nitrates-HMT precursors at ambient air. The general chemical reaction of the synthesis process is described by Eq. 1:

1 $${\left({\mathrm{Co}}_{\mathrm{x}}{\mathrm{Ni}}_{1-\mathrm{x}}\right)\left({\mathrm{NO}}_{3}\right)}_{2}+\left(\frac{1}{3}\varphi \right){{\mathrm{C}}_{6}\mathrm{H}}_{12}{\mathrm{N}}_{4}+3\left(\varphi -1\right){\mathrm{O}}_{2}\to {\mathrm{Co}}_{\mathrm{x}}{\mathrm{Ni}}_{1-\mathrm{x}}+\left(2\varphi \right){\mathrm{CO}}_{2}+\left(\frac{2\varphi +3}{2}\right){\mathrm{N}}_{2}+\left(2\varphi \right){\mathrm{H}}_{2}\mathrm{O}$$

where the *x* values are 1, 0.75, 0.5, 0.25 and 0 for the Co, Co_3_Ni, CoNi, CoNi_3_ and Ni NPs, respectively. φ represents the fuel-to-oxidiser molar ratio. The φ value was for all NMs equal to 1.75, a value shown to be within an optimal range for SCS of pure metals like Co and Ni^33,35^.

**Figure 1:**
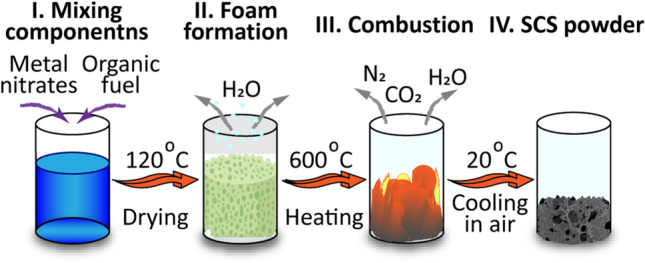
Simplified scheme of the SCS process.

The main synthesis steps are schematically illustrated in Fig. 1.

At first, the required amounts of the metal nitrates were dissolved in a minimal volume of hot distilled water. The HMT was gradually added to the solution under constant stirring at a temperature of 80 °C. After full component dissolution, the solution was dried at 120 °C until gelated and dry foamed powder had been obtained. The foam was then crushed into a powder using an agate mortar and placed in a heat-resistant chemical beaker in a preheated (600 °C) muffle furnace. The synthesis was conducted under normal ambient air atmosphere. The volume SCS reaction started in 20–30 s accompanied by the evolution of a large volume of gases, mostly CO_2_, N_2,_ and vapor. After the precursor combustion, the powder was kept in the furnace for another 5 min for residual carbon burning out. The synthesis temperature was chosen based on previous studies by some of the authors^33,35^, showing the decomposition of initial compounds of the precursor during SCS to be complete at 500 °C. In this case, a temperature of 600 °C was high enough for complete decomposition of the metal nitrates and the fuel, and low enough to prevent metals from intensive oxidation or melting during the SCS reactions. The powder was then removed from the furnace and cooled down to room temperature in a closed beaker to prevent metal oxidation. No inert protection atmosphere was used. Combustion products were then deagglomerated via hand milling in an agate mortar.

### Characterization

The phase composition and crystal structure of the synthesized materials were studied via X-ray diffractometry (XRD) using a Bruker D8 Advance (Germany) instrument with Cu Kα radiation. Diffractogram analysis was made using the HighScore Plus software employing a pseudo-Voigt function for peak profile refinement. The Scherrer equation was used to calculate the size of the crystalline blocks.

Microstructural morphology investigations of the NPs were conducted by means of transmission electron microscopy (TEM) using a Hitachi TEM HT7700 (Japan) microscope and scanning electron microscopy (SEM) using a Hitachi TM-1000 (Japan) instrument, coupled with energy X-ray dispersive spectroscopy (EDS) for elemental analysis. Due to instrumental limitations, the analysis did not include either oxygen or carbon.

The composition of the surface and near-surface regions of the NPs was studied via X-ray photoelectron spectroscopy (XPS) using a Kratos Analytical UltraDLD spectrometer (monochromatic 150 W Al X-ray source on areas sized 700 × 300 µm^2^). Duplicate measurements were performed for each powder. Wide spectra and high-resolution spectra (20 eV pass energy) were acquired for Co 2p and Ni 2p regions, using C 1s as energy reference (285.0 eV). The Shirley method was used to define the baseline and the Gaussian function for peak fitting.

The dry surface area of the samples was determined using a 3Flex analyzer (Micromeritics, USA). Before the measurements, 0.5 g of powder of each powder was degassed for 12 h under vacuum (0.05 mbar) at 300 °C. The specific surface area was calculated by means of the BET method using the 3Flex software.

### Electrochemical corrosion tests

Potentiodynamic polarization curves were acquired in FW and FWN solutions by using a three-electrode method and a Princeton Applied Research multichannel potentiostat. A paraffin-impregnated graphite electrode (PIGE) (tip area of 0.2826 cm^2^) was used as a working electrode onto which ~ 2 mg of NPs were attached by pressing the powders by heating the electrode tip. The electrochemical results were normalized to the surface area of the particles based on the applied mass multiplied by the BET surface area for each type of NP. An Ag/AgCl electrode saturated with KCl was used as the reference electrode and a platinum wire as the counter electrode. Prior to the polarization studies, the PIGE with NPs was stabilized at the open-circuit potential for 6 h. The polarisation potential was determined in a range of at least ± 0.25 V from the OCP value applying a scan rate of 0.17 mV/s.

### Metal release assessment

Metal release studies were conducted in FW and FWN solutions. At first, the metal powder was ultrasonically dispersed in FW or FWN to obtain a suspension with a powder concentration of ~ 1 g/L (suspension#1). The sonication time was 5 min using a microtip and a Branson Sonifier, resulting in 2400 J of delivered acoustic energy^41^. This suspension was diluted to concentrations of 10 mg/L (suspension#2) and ~ 2 mg/L (suspension#3). Suspension #3 was then exposed at 30 °C for 1, 6, and 24 h. After the exposure, a 4 mL sample of suspension #3 was ultracentrifuged for 1 h at 50,000 rpm to precipitate undissolved particles. 3 mL of the supernatant was acidified to a pH < 2 by means of 65% ultrapure HNO_3_ and stored for further studies (sample#1). Another 1 mL of suspension #3 was used to determine the size distribution of the particles in solution by means of the Nanoparticle Tracking Analysis (NTA) method.

Dose samples (sample#2) were prepared from 1.4 mL of suspension #2, acidified using King’s water, and ultrasonication for 4 h. Sample# 2 was used to calculate the fraction of dissolved metals during the metal release (dissolution) test. Total concentrations of Co and Ni in solution were determined by means of graphite furnace atomic absorption spectroscopy (GF-AAS, PerkinElmer Analyst 800 instrument). Background metal concentrations were substantially lower as compared to sample concentrations and, if positive, subtracted. Mean values are reported with standard deviations (shown as error bars) of triplicate independent specimens. All equipment in contact with the solution samples was acid-cleaned using 10 vol.-% HNO_3_ for at least 24 h and rinsed four times with ultrapure water (18.6 MΩ cm). The limits of detection (LOD) were 3.6 µg/L for Co and 2.5 µ/L for Ni. The LOD was based on the standard deviation of the blank, multiplied by three.

Tests with known amounts of Co and Ni added to FW and FWN were performed and analyzed as described above with AAS showing recoveries of 95–100% for both metals. The same procedure was performed to ensure complete NP dissolution in King’s water. These control studies showed recoveries of 95–100%.

The NTA analysis was made using a NanoSightSN300 instrument (Malvern Panalytical, UK). For the experiment, a suspension containing metal NPs was injected into a chamber. In short, a laser beam passes through the chamber and particles scatter light, which makes them possible to track and visualize individually based on their Brownian motion by using a microscope combined with a video camera. Particle size distributions were determined using the NTA software. The hydrodynamic diameters were determined using the Stokes–Einstein equation^42^.

### Adsorption study

The adsorption of different ligands and functional groups from FW and FWN solutions onto the SCS NPs were studied by means of Attenuated Total Reflection—Fourier-Transform Infrared Spectroscopy (ATR-FTIR) using a Bruker Tensor 37 FTIR spectrometer with a platinum ATR–IR accessory. The accessory consists of a diamond crystal with an angle of incidence for the IR beam of 45°. A DTGS detector with a polyethylene window was used for in situ measurements.

15 mg of the SCS NPs was ultrasonically dispersed in 6 mL ethanol for 5 min (see above for details) followed by the transfer of ~ 200 μL of the suspension onto the ATR-IR crystal and left to dry for 2 h at ambient air at room temperature. ATR-FTIR spectra were thereafter collected during the static exposure of the NP-films to the FW and FWN solutions for time periods between 10 min up to 3 h. A total of 256 scans were collected for each time point with a scanning resolution of 4 cm^−1^.

## Results

### Materials characterization

#### SEM, EDS, BET surface area

**Figure 2:**
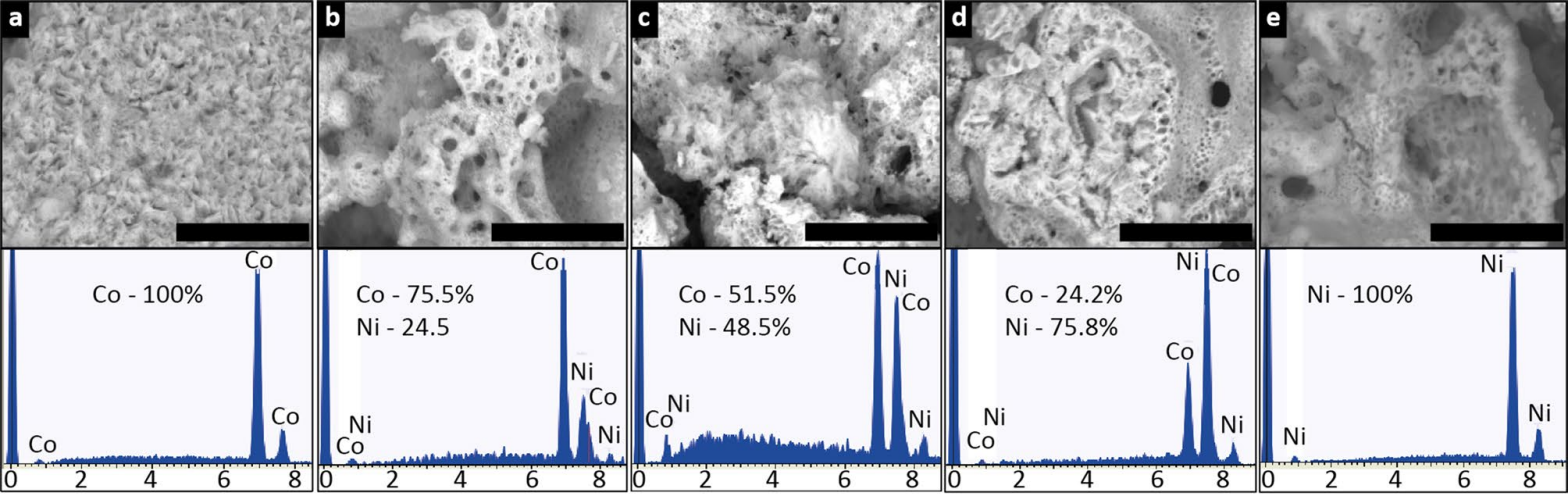
SEM images and results of EDS analysis (at.%) of the (**a**) Co, (**b**) Co_3_Ni, (**c**) CoNi, (**d**) CoNi_3_, and (**e**) Ni NPs. The scale bar equals 10 µm.

Figure 2 shows SEM images of the morphology and elemental composition of the SCS nanopowders.

The images show the SCS nanopowders to consist of both small flakes, which are not aggregated in large grains (Co, Fig. 2a), and non-uniform aggregates with an inhomogeneously distributed porosity (Co_3_Ni (Fig. 2b), CoNi (Fig. 2c), CoNi_3_ (Fig. 2d) and Ni (Fig. 2e), respectively). All NMs have a fine microstructure. In some cases, the grains consist of large compacted aggregates as well as thin porous layers and dendroidal structures, characteristic for SCS powders^43^.

The elemental EDS findings show no other elemental impurities within the Co and Ni metal NPs and the bimetallic NPs to have Co:Ni atomic ratios close to 3:1 (Co_3_Ni), 1:1 (CoNi), and 1:3 (CoNi_3_). Measured BET specific surface areas (*A*) of the NPs were 8.33 m^2^/g (Co), 22.93 m^2^/g (Co_3_Ni), 14.86 m^2^/g (CoNi), 10.53 m^2^/g (CoNi_3_), and 1.01 m^2^/g (Ni). These BET areas are typical for NMs prepared by the SCS approach^36,43^.

#### XRD

**Figure 3:**
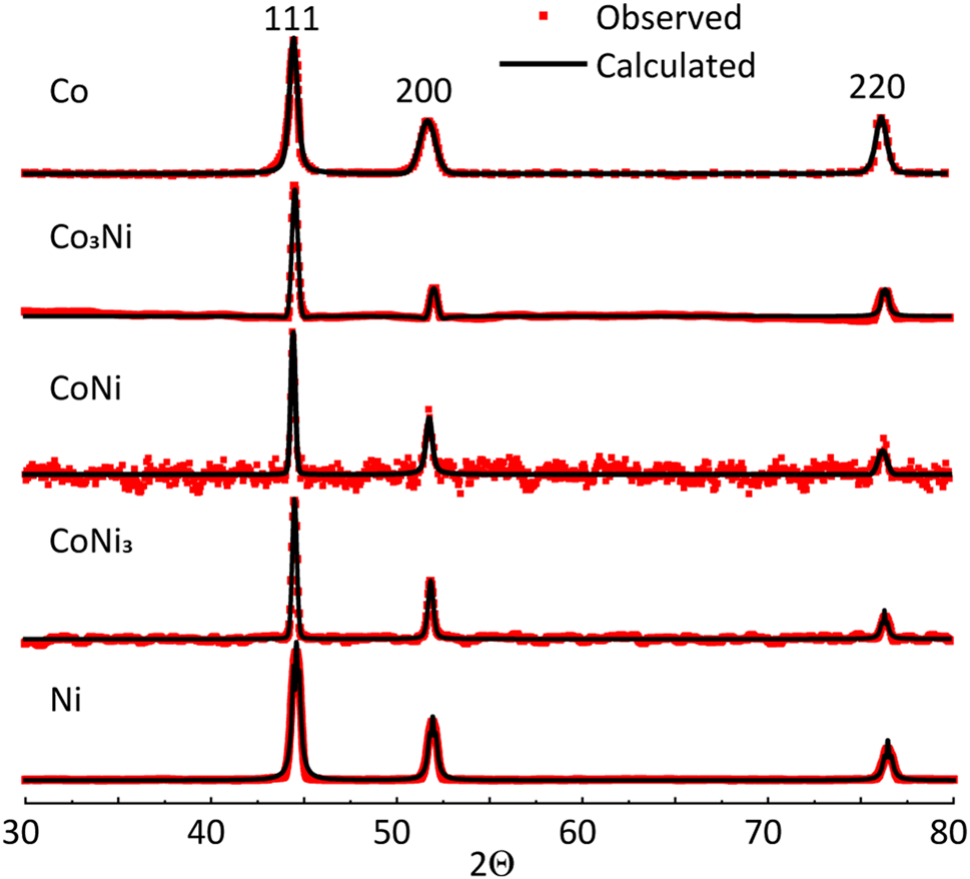
XRD patterns of the Co and Ni-containing NPs.

Typical XRD patterns and calculated crystalline cell parameters (a, V) of the NPs are shown in Fig. 3 and Table 1. According to the XRD data analysis, the NPs consist of single-phase metallic Co, Ni, and bimetallic CoNi NMs with a face-centered-cubic (fcc) crystal structure (Fm-3 m space group). Based on the XRD analysis, no other crystalline phases were observed.

**Table 1:** Calculated crystalline cell parameters and crystalline sizes of the Co- and Ni-containing nanopowders.

NPs	Cell parameter *a*, Å	Cell volume, V, Å^3^	Space group	Crystalline size *d*, nm	Goodness of fit
Co	3.53903	44.32532	Fm-3m (cubic)	11.9	3.53
Co_3_Ni	3.53186	44.05672	Fm-3m (cubic)	21.7	2.11
CoNi	3.53373	44.12663	Fm-3m (cubic)	27.6	7.06
CoNi_3_	3.53118	44.03108	Fm-3m (cubic)	26.4	4.38
Ni	3.52711	43.87920	Fm-3m (cubic)	18.3	0.16

The main (111) peak positions observed at 44.45° and 44.52° in the Co and Ni XRD diffractograms, respectively, were in good agreement with the JCPDS data for Co and Ni metal (PDF: 15-806 Co; 04-0850 Ni). The (111) peak positions observed for Co_3_Ni (44.47°), CoNi (44.46°), and CoNi_3_ (44.48°) were all shifted to a range in-between the peaks for Co and Ni metal. This indicates the formation of distorted bimetallic Co_x_Ni_x−1_ single-phase solid solutions.

The possibility to obtain bimetallic single-phase metallic alloys using the SCS approach has previously been shown by some of the authors^36,44,45^. Calculated values of crystalline sizes (*d*) of the NPs, show all to have a high degree of crystallinity with *d* values ranging from ~ 12 to ~ 28 nm. The variation between triplicate samples was insignificant and could be caused by variations in characteristics of precursors and synthesis parameters.

#### TEM

**Figure 4:**
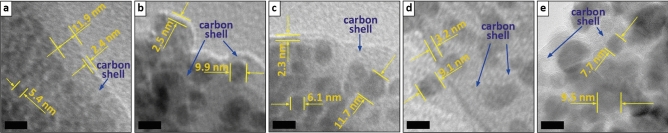
TEM images of the (**a**) Co, (**b**) Co_3_Ni, (**c**) CoNi, (**d**) CoNi_3_ and (**e**) Ni NPs. The scale bar equals 10 nm.

The TEM images of the SCS NPs (Fig. 4 and fig S1) all show the appearance of metal–carbon core–shell structures.

Metallic grains sized between ~ 3 and 16 nm were distributed in a carbon matrix, which covered and separated most grains from each other. The carbon matrix was well crystallized and clearly distinguishable. The Co grains (Fig. 4a and S1a) had the smallest size among all NPs with discrete Co grains typically sized with a diameter of ~ 3.5–4.5 nm. The grains were closely packed in aggregates with a wide carbon border. The NPs of the other NMs were spread in a carbon matrix more inhomogeneously with thicker carbon shells between separate grains (Figs. 4b–e and S1b–e). These structural differences could be related to differences in kinetics of precursor combustion and solid-phase formation. This requires closer investigations and are out of the scope of this study.

#### XPS

**Figure 5:**
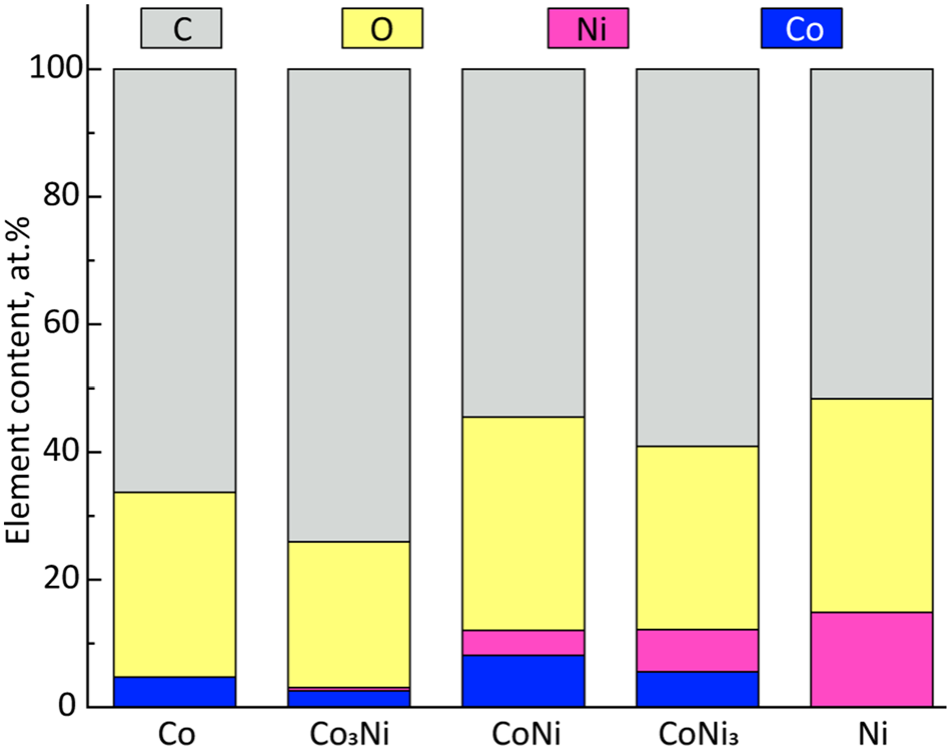
XPS calculated elemental content (at.-%) of the Co- and Ni-containing NPs.

XPS investigations of the SCS powders were carried out for further NPs characterization. Figure 5 and Table S1 show the results of XPS calculated as the average elemental composition of the NPs. Due to the small particle size and the surface information depth of the technique (5–10 nm), the results reflect in addition to the carbon shell, most probably both the surface oxide and to some extent also the core of the NPs. The carbon content exceeded 50 at.% for all powders, reaching the highest value of ~ 74 at.% for the Co_3_Ni powder. All NPs were characterized by high (~ 22–34 at.%) oxygen contents.

Studies of oxidation states of Co and Ni within the outermost surfaces of the metallic NPs were made by analyzing high-resolution XPS spectra of the Co 2p and Ni 2p energy levels (Fig S2). The high noise and low intensity for some of the samples did not allow any unambigously deconvolution of all spectra. However, analysis of results of partially deconvoluted XPS spectra of the Co 2p core level of the metal Co NPs (Figs S2a) and the bimetallic NPs of Co_3_Ni, CoNi, and CoNi_3_ (Fig. S2c,e,g) clearly elucidate the presence of peaks associated with core levels of both Co(III) and Co(II)^46,47^. The XPS spectra of the Ni 2p core level of the Ni NPs (Fig S2b) and the bimetallic Co_3_Ni, CoNi, and CoNi_3_ NPs (Figs. S2d,f,h) show surface oxides consisting of Ni(II)-species^47,48^. Metallic peaks (Co(0), Ni(0)) were observed for the CoNi_3_ NPs at around 778 eV and 852 eV, respectively^47,49^. This implies thinner surface oxides compared to the other NPs.

### Environmental transformations of SCS NPs

#### Nanoparticle dissolution (AAS)

**Figure 6:**
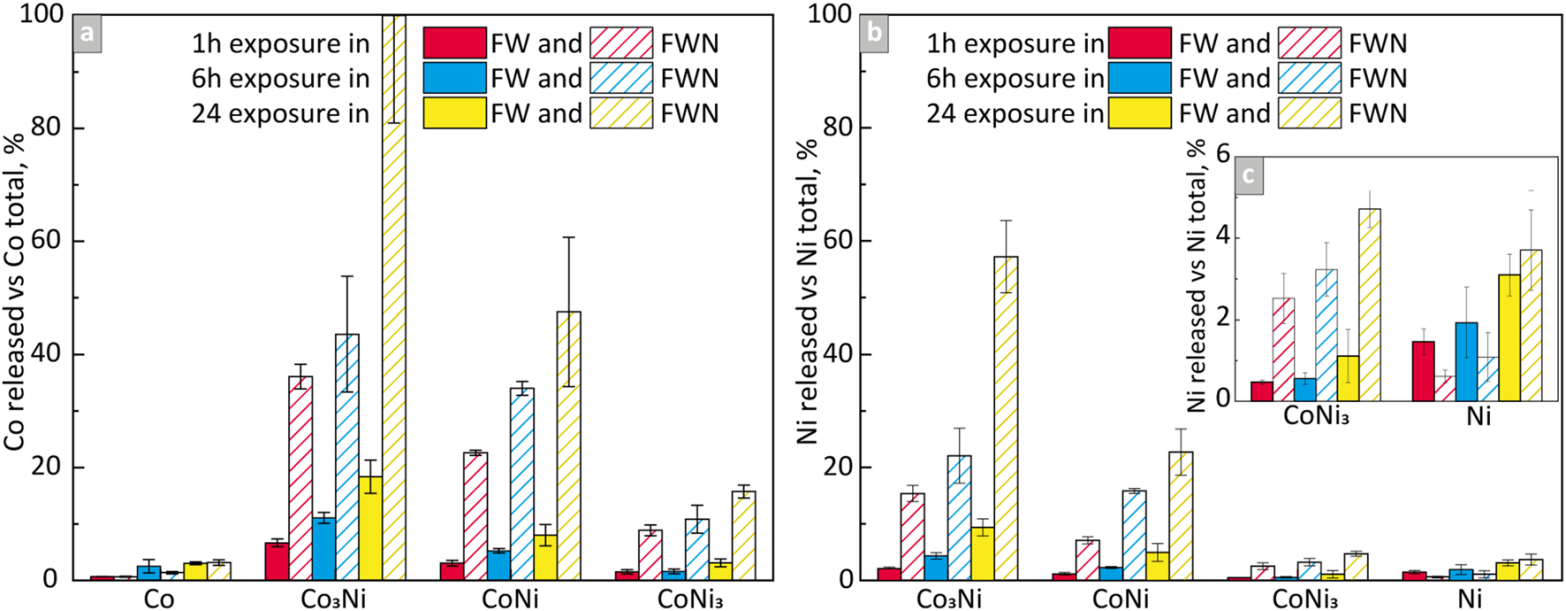
Released amount of (**a**) Co and (**b**) Ni versus the total amount of each metal within the particle loading of the Co- and Ni-containing NPs after 1, 6, and 24 h of exposure in synthetic FW with and without NOM. (**c**) enlarged view of the Ni release from the Ni and CoNi_3_ NPs.

The results of NP dissolution in FW and FWN are presented in Fig. 6 and Fig S3.

More Co was released compared with Ni for all NPs in both solutions and given time points. Most metals (Co + Ni) were released from the Co_3_Ni NPs. The release of Co from the CoNi NPs was ~ 40–54% and ~ 22–54% lower compared with the Co_3_Ni NPs in FW and FWN, respectively. Less Ni was released from the CoNi NPs in FW (~ 50%) compared with FWN (~ 28–60%). The release of Co from the Co and CoNi_3_ NPs, and of Ni from the Ni and CoNi_3_ NPs were ~ 52–90%, ~ 50–85%, ~ 16–67%, and ~ 12–40% (FW) and ~ 93–98%, ~ 61–84%, ~ 84–96%, and ~ 64–92% (FWN) lower compared with both the Co_3_Ni and CoNi NPs. The extent of released Ni from the pure Ni NPs was comparable with the release of Ni from the CoNi_3_ NPs. All NPs showed an increased extent of released metals with time. The release of both Co and Ni was enhanced by the presence of NOM from the bimetallic materials whereas the Co and Ni metal NPs showed a somewhat reduced effect (~ 2–45% and ~ 44–58%, respectively) by the presence of NOM after 1 and 6 h of exposure. No effect was significant after 24 h.

The results of the NTA study of the influence of NOM on the particle size and/or their agglomeration are presented in Fig S4. The sizes of the particle agglomerates in FW were overall larger than in FWN, except for the CoNi_3_ NPs after 24 h. This may indicate a particle stabilizing effect induced by the interaction with NOM, which to some extent hinders the NPs from agglomerating, and which simultaneously enhances the extent of NP dissolution as observed in the dissolution investigations (Fig. 6). However, due to the high standard deviations, which reflect large variations in particle sizes for triplicate samples, it was not possible to reveal any clear trends on the effect of NOM interactions on the mean particle size with increasing exposure time. The most probable reason is related to a too low particle concentration investigated in this study.

#### Adsorption of ligands (ATR-FTIR)

**Figure 7:**
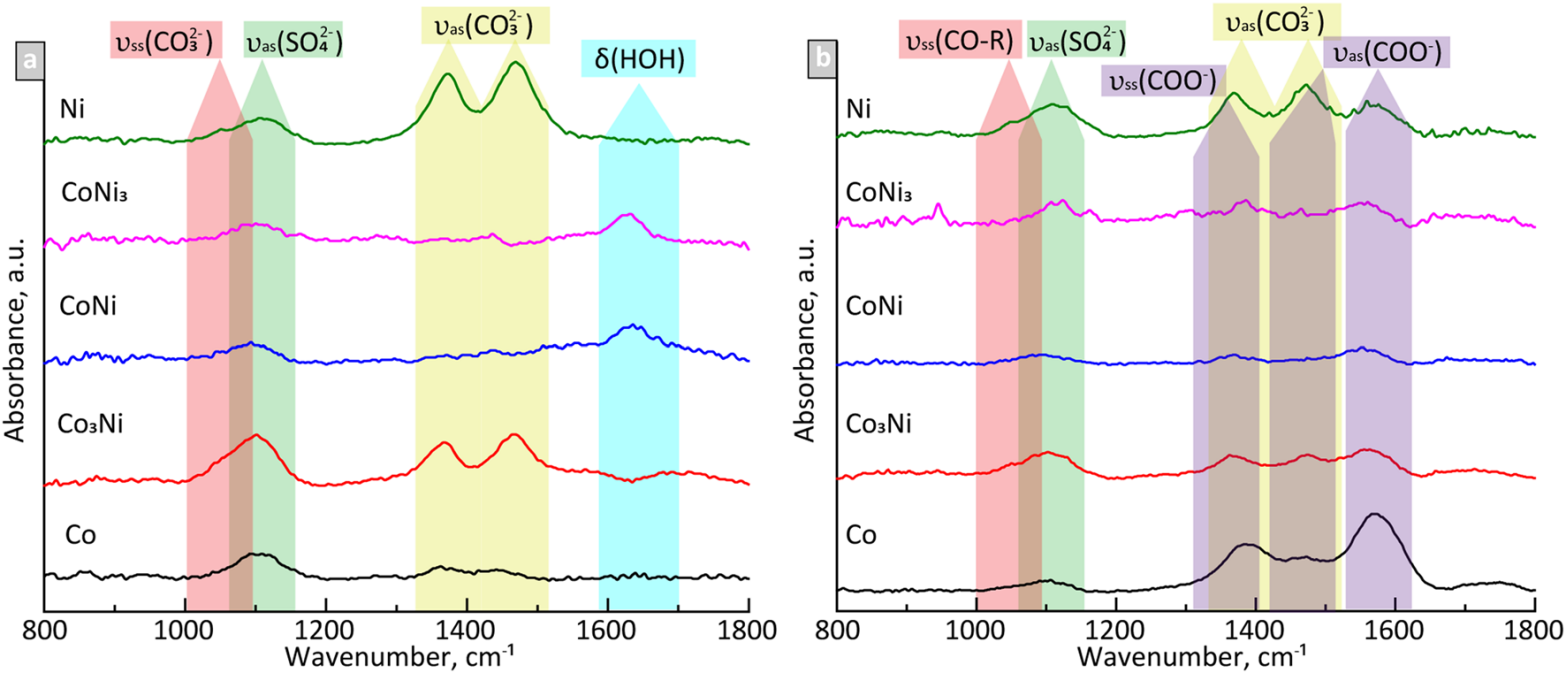
ATR-FTIR spectra of the Co- and Ni-containing NPs exposed for 3 h in (**a**) synthetic FW without NOM and (**b**) FW with NOM.

NOM consists mostly of humic and fulvic acids, which could adsorb onto metal surfaces and, hence, chemically interact with metallic NPs^50^. The nature of this interaction depends on the chemical activity of the metal, its surface charge and pH, as well as on other factors^30^. ATR-FTIR measurements of NPs applied as a layer on the ATR crystal (ca. 1–2 µm thick) were carried out to study the interaction of FW with and without NOM. ATR-FTIR spectra acquired in FW without and with NOM for 3 h are presented in Fig. 7 (spectra at dry conditions are shown in Fig. S5 and kinetic information every 30 min up to 3 h in Fig S6).

According to the data analysis, the spectra of all powders exposed in the FW solution (Fig. 7a), revealed 4 main distinguishable bands. The bands at around 1470–1440 cm^−1^ and 1370–1360 cm^−1^ are both associated with the asymmetric stretching band of carbonate (${\mathrm{CO}}_{3}^{2-}$). The bands at 1115–1090 cm^−1^ and 1060–1040 cm^−1^ are assigned to the sulfate (${\mathrm{SO}}_{4}^{2-}$) asymmetric stretching and the ${\mathrm{CO}}_{3}^{2-}$ symmetric stretching bands, respectively^51^. The vibration peak of bulk water (H_2_O bending mode) at approximately 1680–1630 cm^−1^ was also observed in the spectra of the Co_3_Ni, CoNi, and CoNi_3_ NPs, though lacking for the Co-and Ni NPs. The same bands for the ${\mathrm{CO}}_{3}^{2-}{\mathrm{ and \, SO}}_{4}^{2-}$ groups were observed in the spectra of the NPs exposed in the FWN solution (Fig. 7b). Besides the bands assigned to the asymmetric stretching (1570–1550 cm^−1^ and 1496–1471 cm^−1^) and symmetric stretching (1390–1365 cm^−1^) of the carboxylate group (COO^−^)^52^, CO-R groups (~ 1050 cm^−1^) were also observed in the spectra of all NPs^53^. The bands associated with ${\mathrm{CO}}_{3}^{2-}{\mathrm{ and \, SO}}_{4}^{2-}$, originate from components of the FW solution whereas the COO^-^ band reflects the adsorption of humic or fulvic acid, the main components of NOM. The intensity of the COO^-^ band in the spectra of the Co_3_Ni, CoNi, and CoNi_3_ NPs was substantially reduced compared with the same bands present in the spectra of the Co and Ni metal NPs. As the specific surface areas of the bimetallic samples (see Section "Materials characterization") were significantly larger compared with the Co and Ni NPs, the lower intensities of the COO^-^ bands of the Co_3_Ni, CoNi, and CoNi_3_ spectra may be connected to different electrostatic properties of the surfaces of these powders. Analysis of the time-resolved ATR-FTIR spectra (Fig S6) showed increased peak intensities up to ~ 1.5–2 h of exposure whereafter no intensity change was observed. This indicates maximum adsorption in each case.

#### Electrochemical properties (Potentiodynamic polarization)

**Figure 8:**
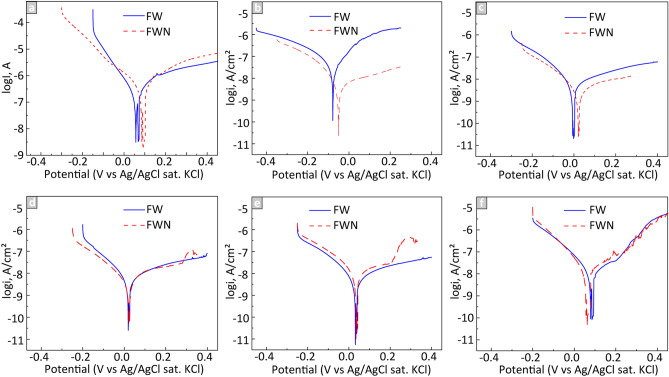
Typical potentiodynamic curves of the (**a**) PIGE, (**b**) Co, (**c**) Co_3_Ni, (**d**) CoNi, (**e**) CoNi_3,_ and (**f**) Ni NPs in synthetic freshwater with and without NOM (FWN, FW).

Potentiodynamic polarization curves of the NPs attached onto PIGE electrodes exposed in FW and FWN are shown in Fig. 8. Observed corrosion potentials (E_corr_) and corrosion current densities (i_corr_) calculated by the Tafel extrapolation method are compiled in Table S2.

The bare PIGE and all NPs exhibited typical cathodic polarization (initial increased negative currents) in the left branch of the polarization curve*.* An increasing potential after E_corr_ leads to a sharp increase in current density, which is indicative of the activation polarization region. After reaching a critical current density value, the activation region passes into the passivation region. Only activation and passivation regions were observed in the anodic polarization branch of the PIGE with Co and Co_3_Ni NPs exposed in both solutions (Fig. 8b,c) and for the CoNi and CoNi_3_ NPs in FW (Fig. 8d,e). The anodic polarization branch of the CoNi and CoNi_3_ NPs in FWN, and of the Ni NPs in both solutions (Fig. 8f) indicates also the presence of a transpassive region, as seen from the sharp increase in current density. It should be noted that the start of the transpassive regions shifted to lower E values as the Ni content increased, from ~ 0.28 V for the CoNi NPs to ~ 0.20 V for the CoNi_3_ NPs and, finally, to 0.18 V for the Ni NPs. This shift, combined with the shift of E_corr_ to a higher potential, resulted for these powders in reduced passivation regions.

Calculations using the Tafel method data indicate lower E_corr_ values and higher i_corr_ values for the Co, Co_3_Ni, CoNi, and CoNi_3_ NPs when exposed in FW than in FWN. At the same time, the E_corr_ was lower for the Ni NPs in FWN compared with FW, even though i_corr_ was still higher in FW. It should be noticed that the E_corr_ and i_corr_ levels systematically changed with increasing concentration of Ni in the powders. E_corr_ increased in the following order: Co < Co_3_Ni < CoNi < CoNi_3_ < Ni. The i_corr_ decreased according to Co > Co3Ni > CoNi and increased following the same sequence for the CoNi_3_ and Ni NPs. The systematic change in the E_corr_ and i_corr_ values indicates a strong connection to the material characteristics as well as interactions taking place with the two solutions.

## Discussion

### SCS NP characteristics

Solution combustion synthesis is a technique in which the main solid phase products form during exothermic redox reactions between components of a precursor, metal nitrates (oxidizer), and organic fuel (reductant), mixed at a molecular level in a solution. This enables combustion of SCS precursors even in an inert atmosphere or in vacuum and results in high-quality NMs with homogeneous crystal phases. After reaction initiation at around 150–250 °C, depending on a precursor, the combustion continues in a sustainable self-propagating or volume combustion mode. In the most common case, no external heat is at this stage required for any further reaction to take place. High-intensive high-temperature volume combustion reactions create conditions for the simultaneous formation of a large number of crystallization centres in a reaction volume. A rapid volume crystallization leads to the formation of highly crystalline solid products with a uniform particle size distribution. Another feature of the SCS process is the evolution of a large volume of hot gases, mostly CO_2_, N_2_, and H_2_O vapor during combustion. Hot gases play an important role in heat transfer in a reacting volume, in promoting faster precursor heating up and final material cooling down, in assisting mixing of components, and influencing the morphology of the materials. Hot gases also create a local inert/reduction atmosphere, which plays a major role in the reduction of metallic components and protects formed metal grains from oxidation.

The general morphological feature of the SCS NPs is the formation of aggregated layers from separate flakes and small grains (Fig. 2). Such morphology forms as a result of a combination of two processes. The first process includes intensive solution drying, during which an increasing precursor viscosity leads to the formation of a highly-porous “solid” foam. The foam becomes a morphology template at the solid-phase crystallization step of the SCS process. The second process is related to gas evolution during combustion. In this case, a sharply increased temperature in the reacting volume leads first to thermal decomposition of the fuel, and secondly to the decomposition of the nitrates. As a local temperature distribution in such a system is uneven, this leads to the formation of a thin shell that consists of already formed solid phases on a surface of an unreacted precursor. The shell prevents gases from free release from the inner layers of the precursor. The gas pressure increases until it exceeds a critical value, defined by the shell strength, after which the shell is destroyed as the result of nano/micro explosions^54^. These processes contribute not only to the powder porosity, but also to material deagglomeration and reduction in crystalline size, which in general lead to an increased surface area of the nanopowders. At the same time, even small features in the combustion of the precursors and phase formation could result in substantial differences in NP morphology and, accordingly, surface area. Another morphology-related important factor which contributes to the surface area of the SCS NPs is the presence of the carbon shell that enfolds the NPs, shown by the TEM (Fig. 4) and XPS (Fig. 5, Table S1) analysis.

The presence of residual carbon is typical for SCS-generated materials. Carbon forms as a result of incomplete fuel burning out and is a result of the dissolution–recrystallization process of carbon atoms on the surface of a metal. Dissolution–recrystallization of carbon has under certain conditions shown to result in the formation of multilayered carbon or graphene shells on metal NPs^23,55^. However, taking into account the presence of oxygen atoms (~ 22–34 at.%, based on XPS findings) at the top surface layers of the NPs, and previous findings of the presence of adsorbed oxygen-containing functional groups on SCS graphene-metal nanocomposites^21^, the surface of the carbon layers of the shell may be functionalized. These functional groups could be adsorbed both during the synthesis process and from the ambient air. At the same time, we assume that the oxygen signal predominantly originates from the thin metal oxide at the surface of the NPs. This assumption was confirmed by high-resolution XPS spectra analysis (Fig. S2) from which we presume that the near-surface metallic layers of the NPs mostly consist of (II)- and (III)-valent oxidized Co (Co NPs) and (II)-valent oxidized Ni (Ni NPs) or their combinations (Co_3_Ni, CoNi, CoNi_3_ NPs) present as CoO/Co(OH)_2_, Co_3_O_4_, NiO, Ni(OH)_2_ and (Co_x_Ni_y_)O_z_, respectively. Oxidation occurs both as a result of the exposure of the NPs in air and due to incomplete reduction of oxides, formed as intermediate products during the SCS process^32,56^. The presence of peaks attributed to Co(0) and Ni(0) in the XRD diffractograms of the Co, CoNi, CoNi_3,_ and Ni (Fig. 3) NPs indicates that these powder particles are covered only with a thin oxide layer on top of unoxidized metal cores.

**Figure 9:**
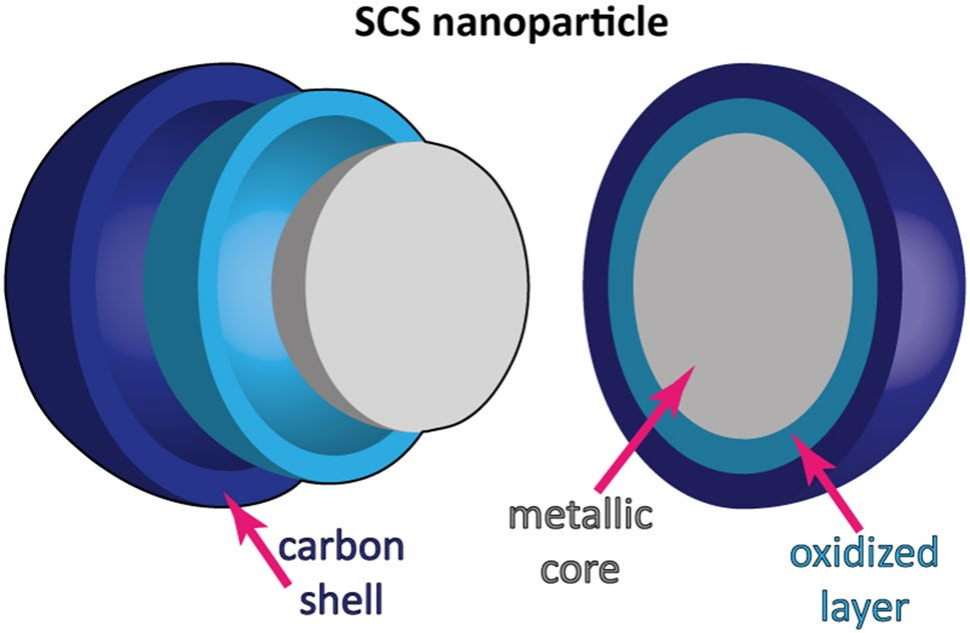
Simplified scheme of the SCS NP structure.

Overall, we can conclude that the synthesized NPs are crystalline and predominantly have mono-phase structures. The NPs have a core–shell structure with thin surface oxides and carbon shells (Fig. 9). The NPs consist of aggregates of grains and porous thin layers. The nature of such core-oxide-shell structures are believed to govern the surface area of the NPs as well as their reactivity and other surface-related properties.

The Co:Ni ratios in the near-surface layers of the NPs were according to XPS analysis shifted to a higher Co content compared with their stoichiometric composition (1.7, 2.1, and 2.5 times higher for the Co_3_Ni, CoNi, and CoNi_3_ NPs, see Fig. 5 and Table S1). This reflects the presence of a Co-rich surface oxide but may also be attributed to inhomogeneities in the outer layers of the precursor/foam, differences in kinetics of phase formation of Co and Ni subphases, and differences in ion mobility at the temperature for the synthesis. Moreover, even though it was not proven experimentally, the phase inhomogeneity in the outer layers of the NPs may lead to a corresponding compensative phase inhomogeneity in the deeper layers. We suppose though that such a layered structure is not representative of the entire volume of the NP, as XRD showed the formation of bimetallic Co–Ni crystal cells without the Co–Ni phase split. The presence of the Co-rich outer surface along with general properties of metallic phases was hypothesized to, at least to some extent, influence the dissolution and corrosion properties of the bimetallic NPs. This is discussed next.

### Dissolution in freshwater and freshwater with NOM

**Figure 10:**
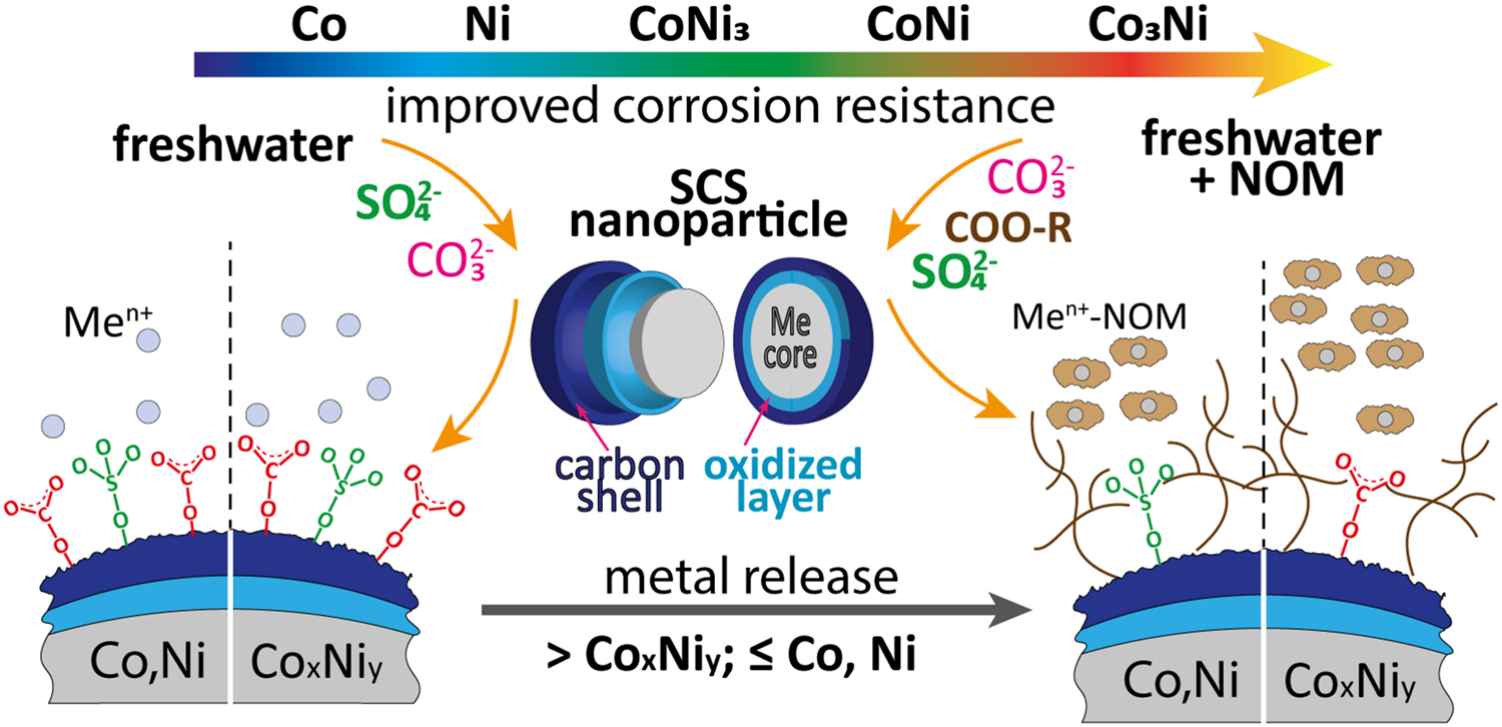
Schematic summary of the results of dissolution and corrosion study of SCS-obtained Co–Ni NPs.

The results of the dissolution and corrosion properties study are schematically summarized in Fig. 10.

According to the release data of Co and Ni from the NPs (Fig. 6) was the total metal dissolution (Co + Ni) higher in FWN than in FW (except after 1 and 6 h of exposure for the Co and Ni NPs). More metal dissolution was observed for the bimetallic NPs (Co_3_Ni, CoNi, CoNi_3_) compared with the pure metal NPs. The electrochemical measurements showed in contrast lower corrosion currents (higher corrosion resistance) after 6 h for the bimetallic NPs compared with the metal NPs, which implies that also chemical processes govern the dissolution of the NPs^57^. The bimetallic NPs showed further slightly higher corrosion currents in FW compared with FWN. These findings were in contrast with the metal release data that showed enhanced release of Co and Ni from the bimetallic NPs in the presence of NOM (Table S2). This effect was not observed for the metal NPs for which the adsorption of NOM resulted in reduced metal release (up to 6 h) followed by no effect after 24 h of exposure.

Complexation between released metals and NOM in solution resulted in a somewhat increased dissolution of the bimetallic NPs but not for the metal NPs. Metal and metal oxide NPs have many times been observed to dissolve faster upon addition of NOM, but there are also examples of the contrary effect^30^. Interestingly is the metallic and bimetallic NPs of this study show opposite results, which indicate slightly different modes of action of NOM with the NP surfaces. As discussed previously, the vibrational modes of NOM were stronger for the metal NPs compared with the bimetallic NPs, which could lead to a higher extent of blocking and hence slower transport of corrosive species to the particle surfaces (Fig. 7).

The higher extent of metal release from the bimetallic NPs compared with the pure metal NPs could be related to their higher BET specific surface areas and thereby more reaction sites for dissolution processes to take place^58^. However, the real surface area in solution is unknown and depends on other aspects such as e.g. agglomeration and hence the fractal dimension of these agglomerates^59^.

The lack of correlation between the electrochemical properties (E_corr_, i_corr_, presence of transpassivity) and the extent of dissolution shows that non-faradaic processes dominate the dissolution of the SCS NPs. ATR-FTIR showed components from the FW solution and of NOM to be physically adsorbed onto the NPs and hence interact in a chemical way on the metal release processes (Fig. 7). Large hydrophobic molecules of NOM could, as previously reported, create a physical protective barrier, preventing direct contact between the surfaces of the NPs and the oxidizing solutions^60^. Adsorption of NOM could also to some extent block the adsorption of carbonate and sulfate (Fig. 7) and thereby prevent them to form any protective surface layers^60^. Similar to the observations of this study the formation of such layers has been observed to result in a reduced corrosion activity. In this case, the formation of more stable and corrosion-resistant Co-rich oxides hindered anodic oxidation and increased the passivation region of the potentiodynamic curve to higher potentials.

We further hypothesized that the composition of the surface oxide can play an important role in the extent of metal release from the bimetallic SCS NPs. The enrichment of Co within the mixed surface oxide correlated with an increased extent of released metals (Fig. 6). These findings could at given conditions be connected to reduced barrier properties of Co oxides (such as CoO/Co(OH)_2_) compared with Ni oxides.

### Connection between corrosion characteristics and mixing entropy of SCS NPs

There is a fundamental interconnection between the mixing entropy of multicomponent single-phase alloys and their mechanical hardness and melting temperature^61^. This interconnection has been used to predict the formation of high-entropy alloys^62^. According to the Boltzmann hypothesis regarding the relationship between the entropy of a system and the system complexity for a random solid solution, the configurational entropy of mixing *ΔS* is represented by Eq. 3:

3 $$\Delta S = - R\mathop \sum \limits_{i} c_{i} ln\,c_{i} ,$$

where *R* is the gas constant (8.314 J/K·mol) and *c*_*i*_ is the molar content of the *i*th component. The *ΔS* value changes from zero, for pure metals, to 0.56R for the Co_3_Ni and CoNi_3_ NPs to 0.69R for the equimolar CoNi NPs, which indicates a higher entropy for equimolar systems.

**Figure 11:**
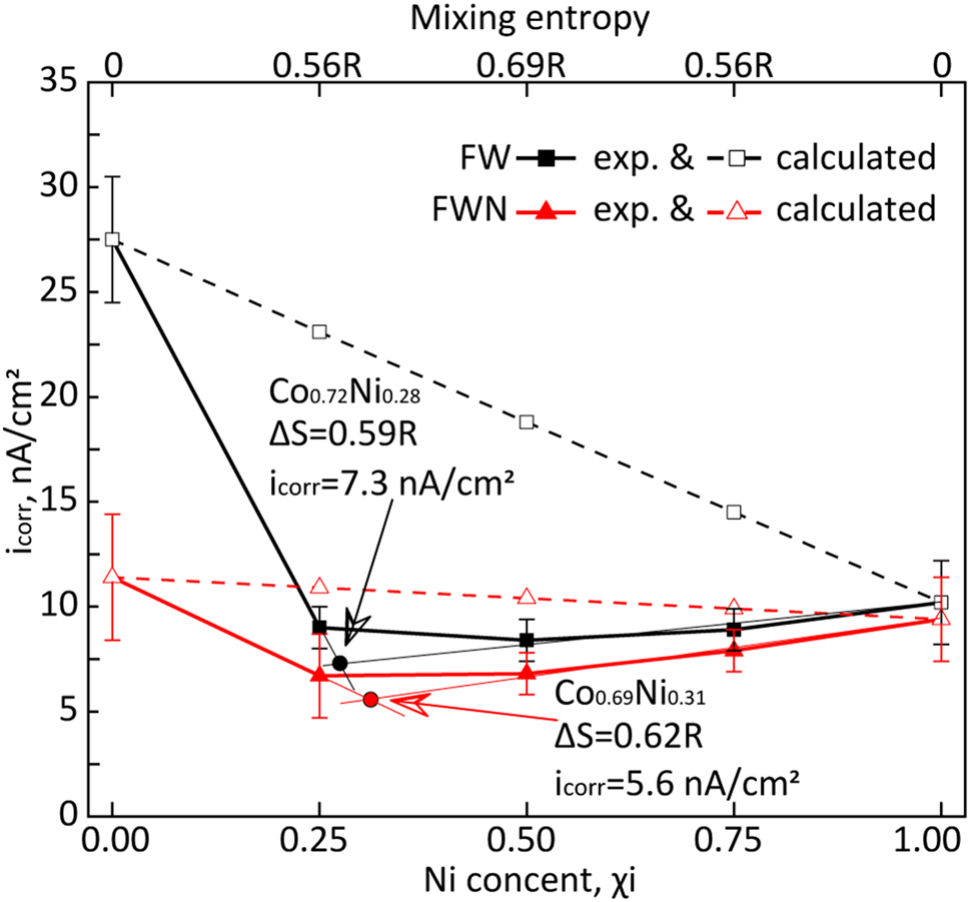
Corrosion current density, i_corr,_ results as a function of Ni content and respective mixing entropy values of the Co- and Ni-containing NPs exposed in FW and FWN solutions. The experimental data is presented as solid lines and the calculated data as dashed lines (via the rule of mixing).

The i_corr_ values determined for the NPs in both FW and FWN are plotted in Fig. 11 (solid lines) as a function of the Ni content of the NPs in order to gain an improved understanding of their corrosion characteristics and elucidate differences in properties between the pure metals and the bimetallic NPs.

The i_corr_ values for the pure metal NPs were higher than corresponding values for the bimetallic NPs and did not follow the simple rule of mixing (Fig. 11, dashed lines). The differences in i_corr_ values between experimental data and data calculated by the mixing rule method were for the Co_3_Ni, CoNi, and CoNi_3_ NPs 61%, 55%, 39% in FW, and 39%, 35%, 20% in FWN, respectively.

The mixing entropy relates to an excess of the system energy, which should be overcome for changing system state—in this case, corrosion. We suppose, like for mechanical and thermal properties, that a higher entropy of the system should lead to the higher corrosion resistance of the metallic NPs. From the i_corr_ data approximation, two compositions in the binary Co–Ni system (Co:Ni molar ratios of 0.72:0.28 and 0.69:0.31) could be the most corrosion resistant materials in FW and FWN solutions with corrosion current densities of 7.3 nA/cm^2^ and 5.6 nA/cm^2^, respectively. Their compositions were characterized by *ΔS* values of 0.59R and 0.62R, which are lower than the theoretically optimal value (0.69R) for an equimolar mixture. This indicates the involvement of other factors, except system entropy, such as valent electron concentration (VEC) and/or formation of stable oxidized outer layers. The VEC parameter is defined as the number of valent electrons per formula unit and has previously been used to predict the stability and properties of binary and trinary compounds and high-entropy materials^63,64^. Conditions with an optimal VEC lead typically to the formation of a material with a layered electronic structure with an uneven reaction on external forces^65^. Since this leads to enhanced mechanical properties of the materials, we suppose that it also could influence the corrosion properties of the NPs. Differences in the composition of the oxide layer could, as discussed above, also influence the corrosion characteristics of the SCS NPs, possibly by reduced ion mobility and electron mobility through the oxide.

## Concluding remarks

Engineered nanomaterials (NMs) obtained by the solution combustion synthesis (SCS) method are complex structures consisting of NPs with metallic cores with oxidized surfaces, and outer carbon shells. The particle morphology and structure depend on the parameters of the synthesis procedure and precursor composition. The dissolution and corrosion properties of SCS-synthesized NMs are interconnected and defined by multiple factors such as phase composition, surface oxide composition, microstructure, specific surface area, and solution chemistry. Results generated for pure and binary SCS NPs of Co and Ni show that the adsorption of large, hydrophobic molecules of NOM could enhance their corrosion resistance (conditions governed by electrochemical processes), and at the same time, via ligand-induced chemical processes (non-faraidic processes), increase their dissolution rates. If environmentally dispersed, transformation/dissolution processes may for such binary NPs, despite improved corrosion properties, result in an increased potency for adverse environmental effects. This emphasizes the importance to characterize and assess nano-specific interactions of functional NMs for different environmental settings and avoid using corrosion resistance data to predict the extent of metal transformation/dissolution.

Further studies of the reactivity, transformation, and dissolution properties of pure and multicomponent metallic compositions of NMs should be performed in order to gain an improved fundamental understanding of the interconnection between the mixing entropy of bi(poly)metallic NMs, their VEC parameters, and their corrosion properties. A combination of experimental studies as described in this study complemented with theoretical modelling of materials state of matter could become a powerful way forward for future predictions of physico-chemical characteristics of bi(poly)metallic NMs.

## Supplementary Information

Supplementary Information
