# Supplementary material for: Corrosion and transformation of solution combustion synthesized Co, Ni and CoNi nanoparticles in synthetic freshwater with and without natural organic matter
Source: Sci Rep. 2021 Apr 12;11:7860. doi: 10.1038/s41598-021-87250-7 (PMC8042015; doi:10.1038/s41598-021-87250-7)
Supplement: Supplementary file 1 — Supplementary Information [file 41598_2021_87250_MOESM1_ESM.docx]

**Corrosion and Transformation of Solution Combustion Synthesized Co, Ni and CoNi Nanoparticles in Synthetic Freshwater with and without Natural Organic Matter**

**(Supporting information)**

Alexander Khort^a,b^*, Jonas Hedberg^a,c^, Nanxuan Mei^a^, Valentin Romanovski^b,d^, Eva Blomberg^a,e^, Inger Odnevall Wallinder^a,f,g^*

^a^KTH Royal Institute of Technology, Department of Chemistry, Division of Surface and Corrosion Science, Stockholm, Sweden

^b^National University of Science and Technology “MISIS”, Research Center of Engineering Ceramic Nanomaterials, Moscow, Russia

^c^Western University, Surface Science Western, London, Canada.

^d^Institute of General and Inorganic Chemistry, National Academy of Sciences of Belarus, Minsk, Belarus

^e^RISE Research Institutes of Sweden, Division Bioscience and Materials, Stockholm, Sweden

^f^AIMES - Center for the Advancement of Integrated Medical and Engineering Sciences at Karolinska Institutet and KTH Royal Institute of Technology, Stockholm, Sweden

^g^Department of Neuroscience, Karolinska Institutet, SE-171 77, Stockholm, Sweden

Corresponding authors e-mails: [khort@kth.se](mailto:khort@kth.se) (AK) and [ingero@kth.se](mailto:ingero@kth.se) (IOW)


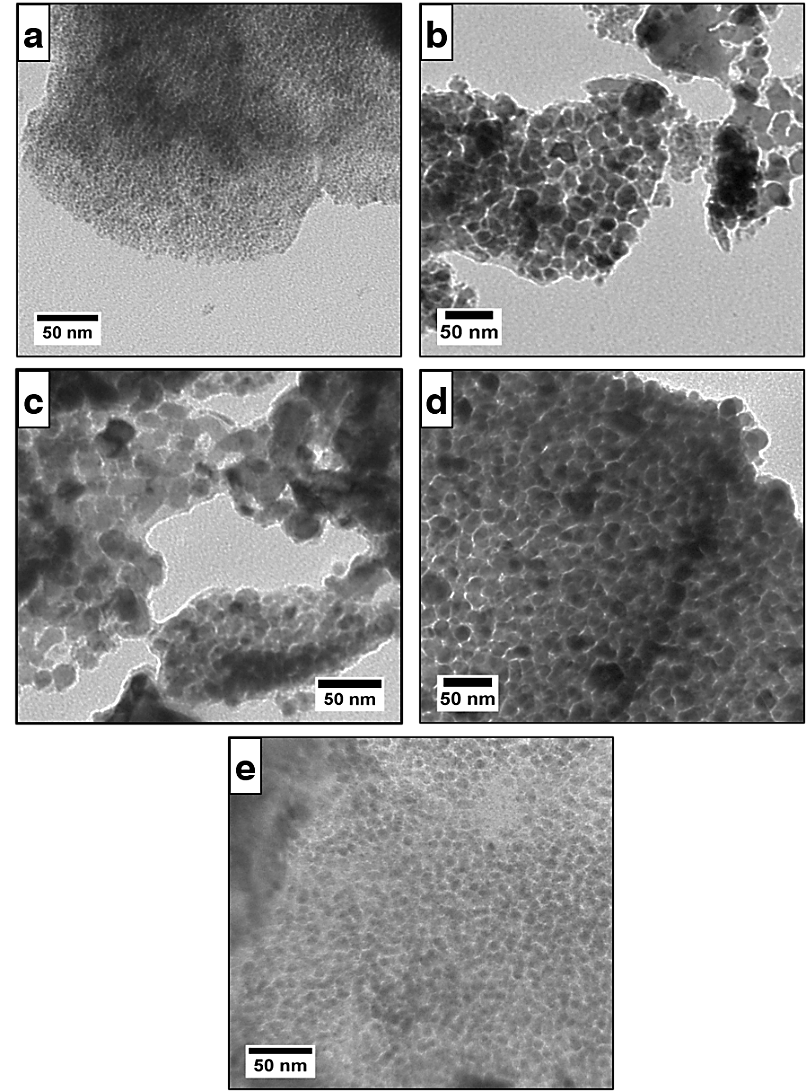


Fig S1. TEM images of (a) Co, (b) Co_3_Ni, (c) CoNi, (d) CoNi_3_ and (e) Ni CSC NPs.

Table S1. – XPS average elemental content in the outermost surface of the Co- and Ni-based NPs.

| Samples | Element content, at.% | | | |
| --- | --- | --- | --- | --- |
|  | Co | Ni | O | C |
| Co | 4.72 | – | 28.98 | 66.30 |
| Co_3_Ni | 2.58 | 0.51 | 22.86 | 74.05 |
| CoNi | 8.12 | 3.91 | 33.47 | 54.50 |
| CoNi_3_ | 5.53 | 6.68 | 28.74 | 59.05 |
| Ni | – | 14.87 | 33.49 | 51.64 |


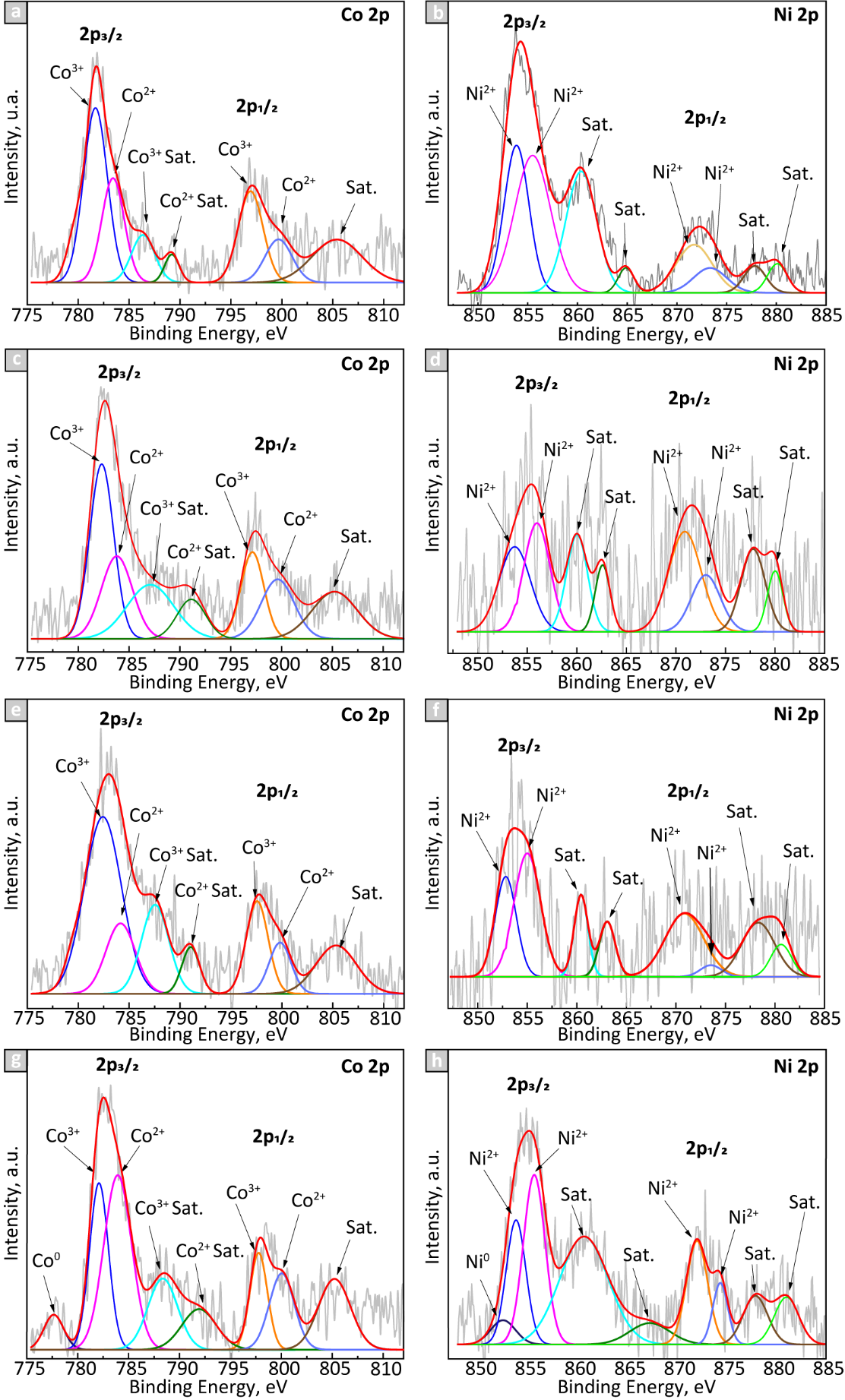


Fig S2 – High resolution XPS spectra of the (a) Co, (b) Ni, (c,d) Co_3_Ni, (e,f) CoNi, and (g,h) CoNi_3_ NPs


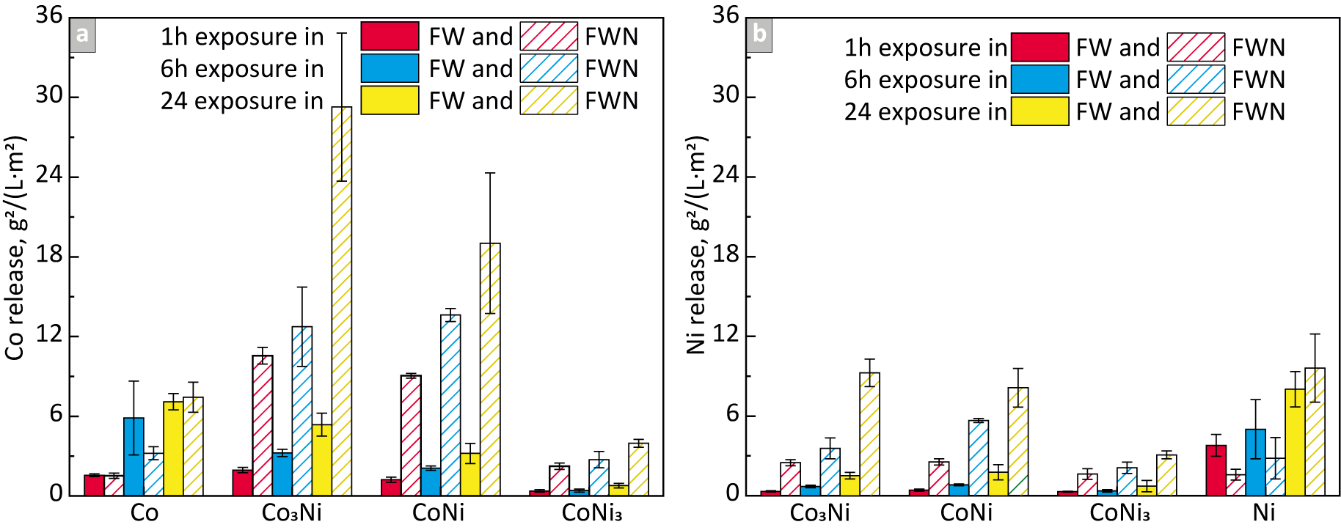


Fig S3 – Surface area normalized amount of release of (a) Co and (b) Ni from the Co- and Ni-containing NPs after 1, 6, and 24 h of exposure in synthetic freshwater (FW) with and without NOM.


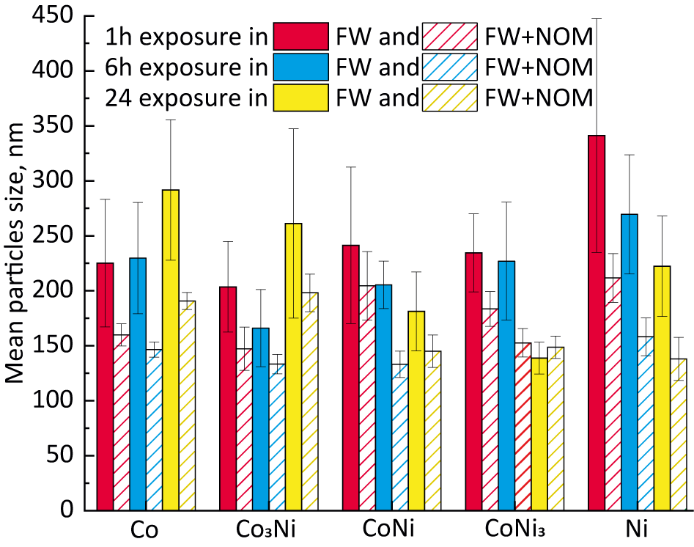


Fig S4. NTA particle size measurements of the experimental samples after 1, 6 and 24 h of exposure in freshwater (FW with and without NOM.


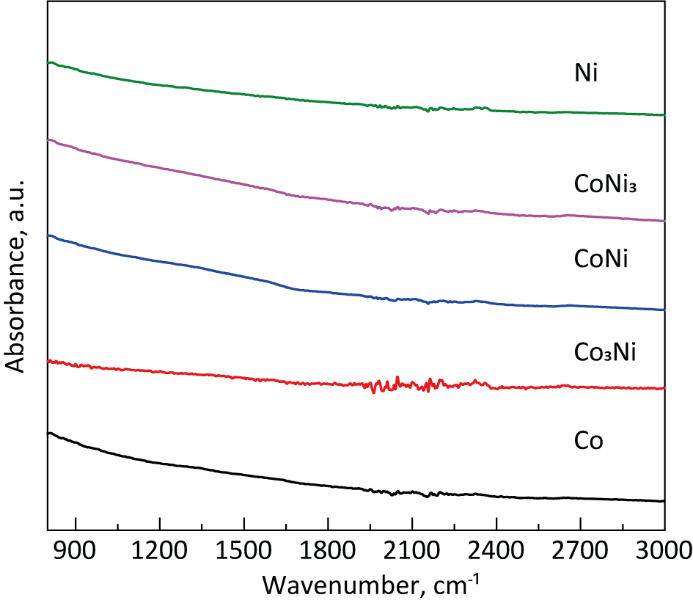


Fig S5 – FTIR spectra of the NPs


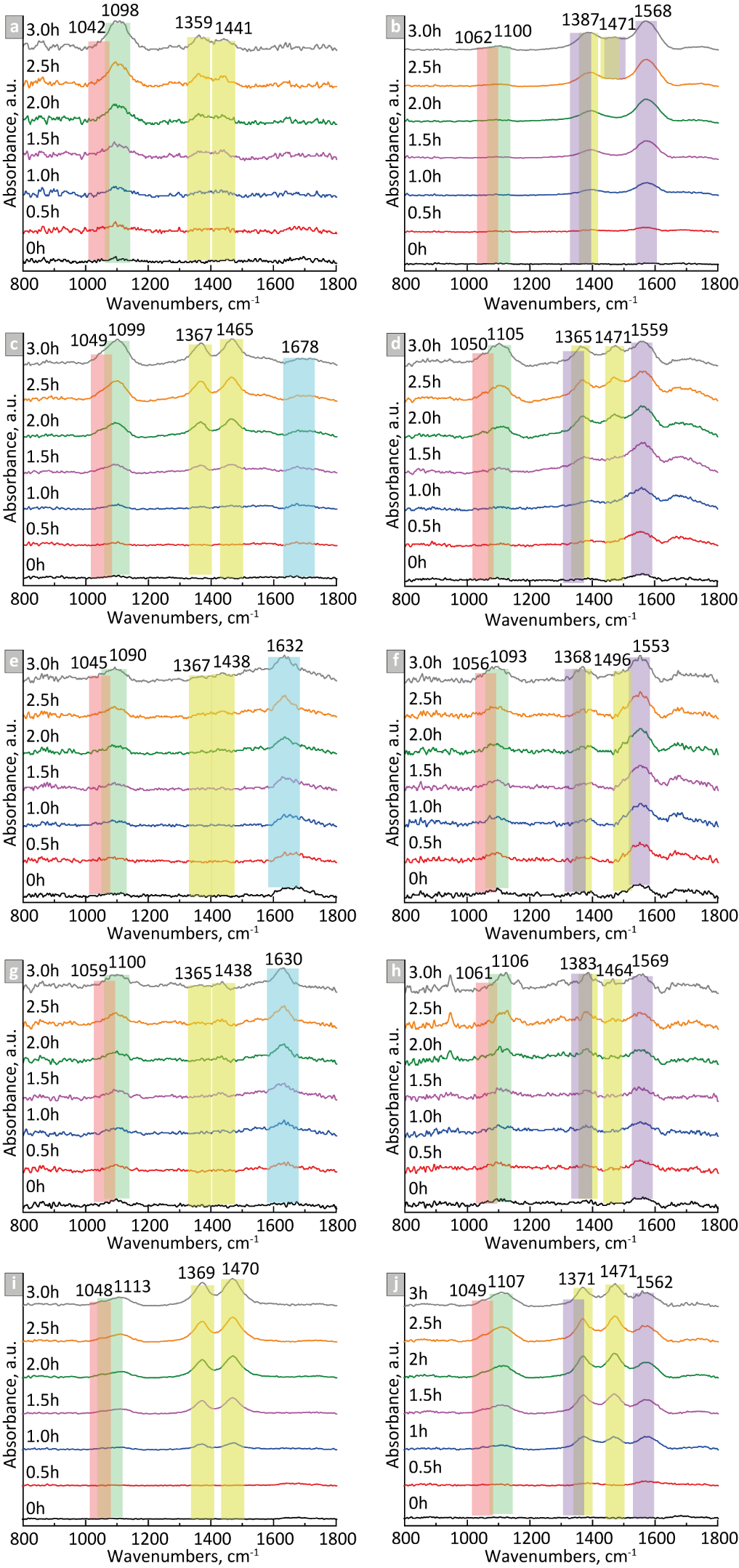


Fig S6. – Time-resolved ATR-FTIR spectra of (a,b) Co, (c,d) Co_3_Ni, (e,f) CoNi, (g,h) CoNi_3_ and (i,j) Ni NPs exposed up to 5 h in FW (left column) and FWN (right column).

Table S2. – Electrochemical corrosion potentials and corrosion currents of the Co- and Ni-based NPs exposed in FW with and without NOM.

| Electrode/NPs | FW | | FW with NOM | |
| --- | --- | --- | --- | --- |
|  | E_corr_, mV | I_corr_, μA/cm^2^ | E_corr_, mV | I_corr_, μA/cm^2^ |
| PIGE | 58.0±3.1 | – | 87.61 | – |
| Co | -77.4±48.4 | 0.0275±0.003 | -50.3±46.2 | 0.0114±0.003 |
| Co_3_Ni | -2.0±3.1 | 0.0090±0.001 | 22.5±5.9 | 0.0067±0.002 |
| CoNi | 19.2±5.2 | 0.0084±0.001 | 24.5±5.7 | 0.0068±0.001 |
| CoNi_3_ | 34.9±4.4 | 0.0089±0.001 | 38.1±4.9 | 0.0079±0.001 |
| Ni | 78.8±5.6 | 0.0102+0.002 | 61.6±8.3 | 0.0094±0.002 |
